# Phosphatidylserine-exposing tumor-derived microparticles exacerbate coagulation and cancer cell transendothelial migration in triple-negative breast cancer

**DOI:** 10.7150/thno.53637

**Published:** 2021-04-19

**Authors:** Cong Zhang, Zhuowen Yang, Peng Zhou, Muxin Yu, Baorong Li, Yingmiao Liu, Jiaqi Jin, Wenhui Liu, Haijiao Jing, Jingwen Du, Jie Tian, Zhiyu Zhao, Jianxin wang, Yinzhu Chu, ChunMei Zhang, Valerie A Novakovic, Jialan Shi, Changjun Wu

**Affiliations:** 1 Department of Ultrasound, The First Hospital, Harbin Medical University, Harbin, China.; 2 Department of Gerontology, The First Hospital, Harbin Medical University, Harbin, China.; 3 Department of Hematology, The First Hospital, Harbin Medical University, Harbin, China.; 4 Department of Research, VA Boston Healthcare System, Boston, MA.; 5 Department of Medical Oncology, Dana-Farber Cancer Institute, Boston, MA.; 6 Department of Medicine, Brigham and Women's Hospital, and Harvard Medical School, Boston, MA.

**Keywords:** Phosphatidylserine, tumor-derived microparticles, procoagulation, transendothelial migration, lactadherin.

## Abstract

**Background:** Neoadjuvant chemotherapy is relevant to the formation of thromboembolism and secondary neoplasms in triple-negative breast cancer (TNBC). Chemotherapy-induced breast cancer cell-derived microparticles (BCMPs) may have important thrombogenic and pro-metastatic effects on platelets and endothelium, which may be related to the expression and distribution of phosphatidylserine (PS). However, investigating these interactions is challenging due to technical limitations.

**Methods:** A study was conducted in 20 healthy individuals and 18 patients who had been recently diagnosed with TNBC and were undergoing neoadjuvant chemotherapy with doxorubicin and cyclophosphamide. BCMPs were isolated from patient blood samples and doxorubicin-treated breast cancer cell lines. Their structure and morphology were studied by electron microscopy and antigen levels were measured by fluorescence-activated cell sorting. In an inhibition assay, isolated BCMPs were pretreated with lactadherin or tissue factor antibodies. Platelets isolated from healthy subjects were treated with BCMPs and coagulation time, fibrin formation, and expression of intrinsic/extrinsic factor Xase (FXa) and thrombin were evaluated. The effects of BCMPs on endothelial thrombogenicity and integrity were assessed by confocal microscopy, electron microscopy, measurement of intrinsic/extrinsic FXa, prothrombinase assay, and transwell permeability assay.

**Results:** Neoadjuvant chemotherapy significantly increased the expression of PS+ BCMPs in patient plasma. Its expression was associated with a rapid increase in procoagulant activity. Treatment with lactadherin, a PS-binding scavenging molecule, markedly reduced the adhesion of BCMPs and abolished their procoagulant activity, but this was not observed with tissue factor antibody treatment. Intravenous injection of BCMPs in mice induced a significant hypercoagulable state, reducing the extent of plasma fibrinogen and promoting the appearance of new thrombus. Cancer cells incubated with doxorubicin released large numbers of PS+ BCMPs, which stimulated and transformed endothelial cells into a procoagulant phenotype and increased the aggregation and activation of platelets. Moreover, cancer cells exploited this BCMP-induced endothelial leakiness and showed promoted metastasis. Pretreatment with lactadherin increased uptake of both PS+ BCMPs and cancer cells by endothelial cells and limited the transendothelial migration of cancer cells.

**Conclusion:** Lactadherin, a biosensor that we developed, was used to study the extracellular vesicle distribution of PS, which revealed a novel PS+ BCMPs administrative axis that initiated a local coagulation cascade and facilitated metastatic colonization of circulating cancer cells.

## Introduction

Pathological hemostatic system activation occurs in epithelial malignancies 1, 2 such as triple-negative breast cancer (TNBC), which comprises 15-20% of breast cancers. TNBC has no approved molecularly targeted therapy 3-5. Thus, given the invasive biology and high risk of distant recurrence, systemic chemotherapy is warranted in TNBC patients 6. Disappointingly, the frequency of venous thromboembolism (VTE) in breast cancer patients receiving chemotherapy (adjuvant/neoadjuvant) was found to be 4-5 times higher than that in control patients 7-9. The incidence of VTE remains significantly higher with chemotherapy even though current guidelines recommend application of VTE thromboprophylaxis in high-risk individuals 10, 11. This leads us to believe that there are other mechanisms underlying cancer-related VTE in the condition of chemotherapy.

Standard dose-dense doxorubicin (DOX) and cyclophosphamide neoadjuvant chemotherapy (NAC) benefits women with TNBC by downsizing the tumor and increasing pathological complete response rates. However, the therapeutic benefits of NAC may be limited by tumor-promoting host responses that are induced by certain cytotoxic drugs. Several studies have reported pro-metastatic effects of DOX in mouse mammary tumor models 12, 13. Clearly, the NAC per se may be relevant to the formation of thromboembolism and secondary neoplasms. Speculating on the conditions and specific mechanisms of this process is conducive to the development of new targeted anticoagulants or cancer drugs.

Matsumura et al. reported subtypes of tumor cell-derived extracellular vesicles having differently externalized phosphatidylserine (PS) 22. The location of PS on membranes is important for tumor cell survival, growth, and proliferation and cancer-related symptoms 23. However, relatively little is known about the modulation, kinetics, and role of PS^+^ tumor-derived microparticles (MPs) in cancer-associated thrombosis. In the past, special focus was given to tissue factor (TF)-expressing tumor-derived MPs 24-30. But exposed TF is generally quiescent unless residing in a PS^+^ membrane. Thus, TF activity can be inhibited by blocking inhibitors of PS, such as annexin V or lactadherin 31-35. Methodologically, previous studies using thrombin generation assays and transmission electron microscopy (TEM) to analyze the upregulation of procoagulant activity (PCA) 36 could not identify or localize the expression of specific bioactive proteins at the MP level, nor could the procoagulant heterogeneity of MPs on platelets be studied. Additionally, studies may also be needed on the production of intrinsic factor Xase (FXa) and extrinsic FXa activity, including tests of whether this activity can be decreased by blocking exposed TF or PS. A definitive role for the modulation of PS expression and breast cancer cell-derived MPs (BCMPs) arrest and elimination by endothelial cells (ECs) in the process of NAC remains unknown, although interactions between MPs and endothelia are in the spotlight 37-39.

Here, we examined the procoagulant properties and pro-metastatic potential of tumor-derived MPs *in vitro* and *in vivo*. PS^+^ BCMPs were more highly expressed than TF^+^ BCMPs and were complexed to factor Va (FVa) and FXa, which augmented their ability to promote fibrin formation and increased the incidence of microvascular obstruction in vital organs. Moreover, PS^+^ BCMPs induced functional and morphological abnormalities associated with platelets and endothelium, thereby worsening the hypercoagulability state and cancer cell transendothelial migration in both dose- and time-dependent manners. Blocking PS enabled us to delineate the relative contribution of BCMPs to thrombosis and transendothelial migration. PS (found on the surface of cancer cells)-targeted cancer therapy is already well established through PS antibodies and cationic liposomes entrapping DOX 40, 41. Lactadherin, which specifically targets PS on tumor cells and tumor-derived MPs and senses the quantity of exposed PS, in combination with other cancer drugs merits further investigation.

## Materials and methods

### Patients

**Figure 1:**
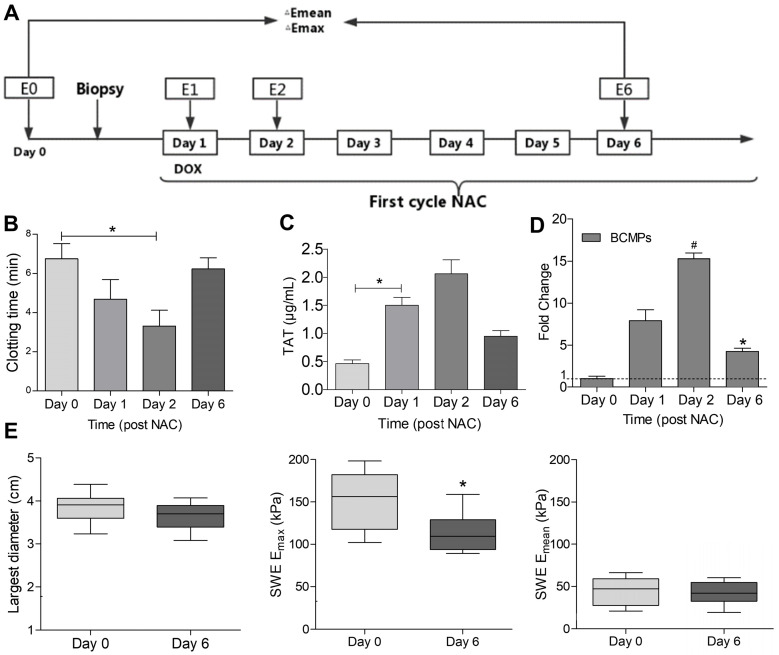
** Clotting time and TAT in patients with TNBC undergoing NAC.** (A) Flow chart of the study design. Peripheral venous blood was obtained on days 0, 1, 2, and 6 in the first NAC cycle. Stiffness parameters derived from shear wave elastography (SWE) images were also measured at the same timepoints (E0, E1, E2, E6). Percentage changes in maximum elasticity (E_max_) and mean elasticity (E_mean_) relative to baseline (ΔE) were calculated on days 1, 2, and 6. (B) Clotting time of whole blood. (C) TAT level in serum. (D) Fold change in MUC1^+^ MPs. (E) Largest diameter, E_max_, and E_mean_ of tumors. ^*^P < 0.05, ^#^*P* < 0.01 vs. Day 0 by Student's *t*-test. Abbreviations: BCMPs, breast cancer cell-derived microparticles; DOX, doxorubicin; NAC, neoadjuvant chemotherapy; TAT, thrombin-antithrombin complex.

The study was conducted between December 2016 and July 2018 with 20 healthy individuals, who were not on any medications two weeks prior to the study, as control and 18 patients who had been recently diagnosed with TNBC and were undergoing NAC with DOX and cyclophosphamide. The test participants were included based on their pretreatment analysis, which was done through pathological examination including HER2 and tissue hormone receptors status. All test patients were treated with DOX and cyclophosphamide according to the recommended guidelines. Tumor stiffness for each patient was assessed 1 day before biopsy (time point Day 0, elasticity E0) and 1, 2, and 6 days after (E1, E2, E6). Peripheral venous blood was withdrawn from patients at the same timepoints during the first NAC cycle (Figure 1A). Further relevant criteria for eligibility were normal cardiac function (left ventricular ejection fraction of 55%), no evidence of distant disease or known or suspected cardiac disease, no diabetes, liver problems, or renal dysfunctions, no previous thromboembolic event, no known hemorrhagic diathesis or coagulopathy, no major surgery within the past 28 days or anticipation of the need for major surgery during the study, and no concurrent treatment with other anti-coagulant, anticancer, or investigational agents 4. The study was performed in accordance with the Helsinki Declaration and was approved by the Ethics Committee of Harbin Medical University.

### Reagents

Bovine serum albumin (BSA), DOX, and ethylenediaminetetraacetic acid (EDTA) were procured from Sigma-Aldrich (MO, USA). 4',6-diamidino-2-phenylindole (DAPI) was purchased from Beyotime (Shanghai, China). Alexa Fluor 488- or 647-conjugated lactadherin or annexin V and fluorescein-labeled fibrinogen were prepared in-house. Human factors prothrombin, Va, VIII, IXa, X, and Xa and thrombin were procured from Haematologic Technologies (VT, USA). Monoclonal antibodies against CD31, CD41a, CD142, and mucin 1 (MUC1) were procured from Becton Dickinson Biosciences (CA, USA). The human TNBC cell line MDA-MB-231 and MCF-7 was a gift from Dr. James O'Kelly (CA, USA). Anti-VE-cadherin, human umbilical vein endothelial cells (HUVECs), EC growth medium, and poly-L-lysine were purchased from Abcam (Stamford, CT). Anti-β-actin antibodies were obtained from Sigma-Aldrich (St. Louis, Missouri). Methamphetamine (TRITC)-phalloidin and fluorescein isothiocyanate-phalloidin were purchased from Shanghai Yu Sheng (Shanghai, China). Fetal bovine serum was obtained from Gibco (NY, USA).

### Cell preparation and MPs collection

Peripheral blood was extracted using a needle (21 gauge) and collected at 4 timepoints (Figure 1A) in a 5 mL tube with sodium citrate (3.8%). After collection, all specimens were centrifuged at 200 × *g* at room temperature for ~15 min. Platelet-rich plasma was extracted and further diluted with modified Tyrode's buffer (137 mM NaCl, 1 mM MgCl_2_, 2.7 mM KCl, 11.9 mM NaHCO_3_, 5.5 mM glucose, 5 mM HEPES, 0.42 mM NaH_2_PO_4_, 0.35% BSA, pH 7.4). The specified Tyrode's buffer was supplemented with 1 mM EDTA. After extracting the plasma, the platelets were centrifuged at 1000 × *g* for 5 min and resuspended in Tyrode's buffer. Platelet count was measured using a blood cell counter.

MPs were isolated following previously described methods 20. The blood samples were centrifuged at 1500 × *g* for 20 min at room temperature. The upper layer of plasma was extracted and subjected to centrifugation at 13000 × *g* for 2 min in order to get rid of residual platelets. Platelet-free plasma (PFP) was obtained, snap-frozen in liquid nitrogen, and then stored at -80 ºC until use. To isolate MPs, 250 μL of cryo-stored PFP was thawed on ice for 1 h and centrifuged at 20,000 × *g* for ~45 min at 20 ºC. Subsequently, 225 μL of supernatant, which was practically free of MPs, was discarded. The remaining 25 μL of the pellet was washed once with Tyrode's buffer, centrifuged, and resuspended in Tyrode's buffer for flow cytometry to assess the source and quantity of MPs.

BCMPs were isolated from conditioned media from confluent MDA-MB-231 and MCF-7 cells by differential centrifugation, as previously described 42-44. The control cells were grown over with serum-free medium (SF) without any additional supplements. MDA-MB-231 and MCF-7 cells were resuspended at 5 × 10^5^ cells/mL and then treated with 0.5, 1, or 2 μM of DOX for 24 h at 37 °C. The supernatant was collected and centrifuged at 1000 × *g* for ~10 min, then at 14,000 × *g* for 2 min at 4 ºC, and again at 14,000 × *g* for 60 min. Through the first and second centrifugation steps, floating impurities were removed, whereas the third centrifugation step sedimented the MPs. The pellets were washed three times with phosphate-buffered saline (PBS) and further resuspended in either PBS or SF. The obtained BCMPs were stored at -80 ºC until further use.

### FVa/FXa binding and fibrin formation on BCMPs and cultured cells

As described previously, to observe the contribution of BCMPs to fibrin formation, BCMP suspensions (2.5 × 10^4^ or 5.0 × 10^4^/μL) were incubated with prewarmed MP-depleted plasma (15%) in the presence of 3 mM of calcium. Fibrin networks were imaged by scanning electron microscopy (SEM) and laser confocal microscopy in the presence of Alexa Fluor 647-conjugated fibrin antibody. Background signal was calculated using a similarly labeled isotype-matched control antibody. Cells and nuclei were visualized by actin staining with fluorescein isothiocyanate-phalloidin (1 μg/mL) and DAPI (300 nM), respectively. BCMPs were stained with Alexa Fluor 488-labeled lactadherin and Alexa Fluor 647-labeled annexin V and imaged. In addition, fibrin formation was quantified by turbidity as previously described 45. Isolated BCMPs or cultured platelets/ECs were added to re-calcified (10 mM, final) MP-depleted plasma (88%, final) in the absence or presence of 128 nM lactadherin or 25.6 μg/mL TF antibody. Fibrin formation was measured by turbidity at 405 nm using a SpectraMax 340 PC plate reader.

### Rate of fibrinolysis

Fibrin clots were formed as described above with some modifications 46. Plasminogen was added to the fibrinogen before addition of thrombin and either tissue plasminogen activator (tPA) or urokinase plasminogen activator in the presence or absence of BCMPs. Because the turbidities were far higher in the presence of BCMPs, data were normalized based on maximal turbidity.

### Clotting times of BCMPs and platelets

A one-stage recalcification assay was carried out to measure the clotting times of platelets and BCMPs and was analyzed further using a STart4 coagulometer (Diagnostica Stago) 21. To complete the assay, 100 µL of cell suspension in Tyrode's buffer was incubated in 100 µL of PFP for ~3 min at 37 ºC. The cells were activated using 100 µL of warmed CaCl_2_ (1.5 mM, final) and clotting time was measured.

### Tail bleeding test

Tail bleeding time was measured in adult (3-4 months old) male and female wildtype mice (Beijing Vital River Laboratory Animal Technology Co. Ltd) as described previously 48 with some modifications. The animal experiments were approved by institutional ethics. After anesthesia, a standardized incision was made on the central dorsal tail vein and then the tail was immersed in PBS at 37 ºC. The time was recorded from the moment blood was observed to emerge from the wound until cessation of blood flow.

### EC reconstitution experiments

Sequential stimulation of HUVECs was achieved until confluency using EC growth medium. Once confluent, the ECs were stimulated with isolated BCMPs at 2.5-5.0 × 10^4^/μL for 8, 16, and 24 h at 37 °C. After incubation, the cells were immunostained using previously described procedures with some modifications 49. Adhesion junctions were stained using an anti-VE-cadherin stain. HUVECs were seeded on coverslips precoated with fibronectin at a seeding density of 3 × 10^5^ cells/coverslip. Cellular confluency was achieved after 4-6 days through addition of EC growth basal medium with BCMPs. After stimulating the BCMPs, the cells were gently washed with PBS once and fixed using 4% paraformaldehyde for ~15 min. The cells were permeabilized with 0.1% Triton X-100 for ~15 min. After permeabilization, 2% BSA was added as a blocking buffer for ~30 min. The samples were incubated overnight at 4 ºC with VE-cadherin antibodies at a dilution of 1:100. The cells were washed three times with PBS and incubated for 1 h at room temperature with Alexa Fluor 488-conjugated mouse anti-rabbit antibodies at a dilution of 1:200. The cells were stained with TRITC-phalloidin (5 µL of methanol stock solution in 200 µL of PBS) for 10 min and DAPI for another 5 min. The slides were mounted using ProLong Gold Antifade Reagent (Shanghai, China). Each test was performed in triplicate.

### Additional methods

The methods for flow cytometry, SEM, TEM, confocal microscopy, platelet isolation from human blood, assays for extrinsic and intrinsic FXa and prothrombinase activity (following previously described methods 47), and assays for thrombin-antithrombin complex (TAT), endothelium permeability, and EC barrier function are presented in detail in Supplementary Methods.

### Statistical analysis

Data is presented as mean ± standard deviation (SD) of at least triplicate measurements. Statistical analysis was performed with Student's *t*-test or ANOVA, as appropriate. *P* < 0.05 was statistically significant.

## Results

### Expression of BCMPs and PCA enhancement in patient plasma after NAC

The characteristics of the patients before NAC are listed in Table 1. In comparison with the control group, the breast cancer group exhibited increased prothrombin time (PT) and activated partial thromboplastin time (aPTT) and increased levels of fibrinogen and D-dimer (Table 1). Further, we measured the PCA of the patients 0, 1, 2, and 6 days after NAC, as depicted in Figure 1A. The clotting time was considerably shorter on days 1 and 2 but returned to baseline on day 6 (Figure 1B). In contrast, the level of TAT in the plasma significantly increased on days 1 and 2 and decreased on day 6 (Figure 1C). BCMP marker expression greatly increased on days 1 and 2 and reduced considerably on day 6 (Figure 1D). These clinical observations indicate a potential relationship between BCMP function and the procoagulation system. Ultrasound is an important tool for assessing the efficacy of NAC in TNBC. NAC-induced tumor cellular apoptosis is indirectly supported by evidence of tumor shrinkage (Fig 1E, left), a drop in tumor stiffness (Fig 1E, middle and right), and improvements in symptoms.

### Elevated PS exposure, BCMP generation, and PCA of BCMPs

Changes in the PCA of BCMPs within the first cycle of NAC were evaluated on days 0, 1, 2, and 6. Flow cytometry analysis with lactadherin and MUC1 antibody further revealed the expressions of PS and MUC1, respectively (Table 2). These results further confirmed that the level of BCMPs on day 1 was elevated 10-fold and was highest on day 2 (Figure 1D). This led us to believe that patients express high levels of BCMP-associated PCA at 24 h after NAC, which is significantly reduced within one week. This profile is associated with prothrombic state as a key indicator, with BCMPs playing a crucial role. In addition, a relatively limited number of TF^+^ MPs and abundant PS^+^ MPs were observed within the first cycle (Table 2). To further explore their function, we simulated an *in vivo* chemotherapy environment to obtain BCMPs and conducted *in vitro* and *in vivo* experiments.

### Collection and characterization of BCMPs

**Figure 2:**
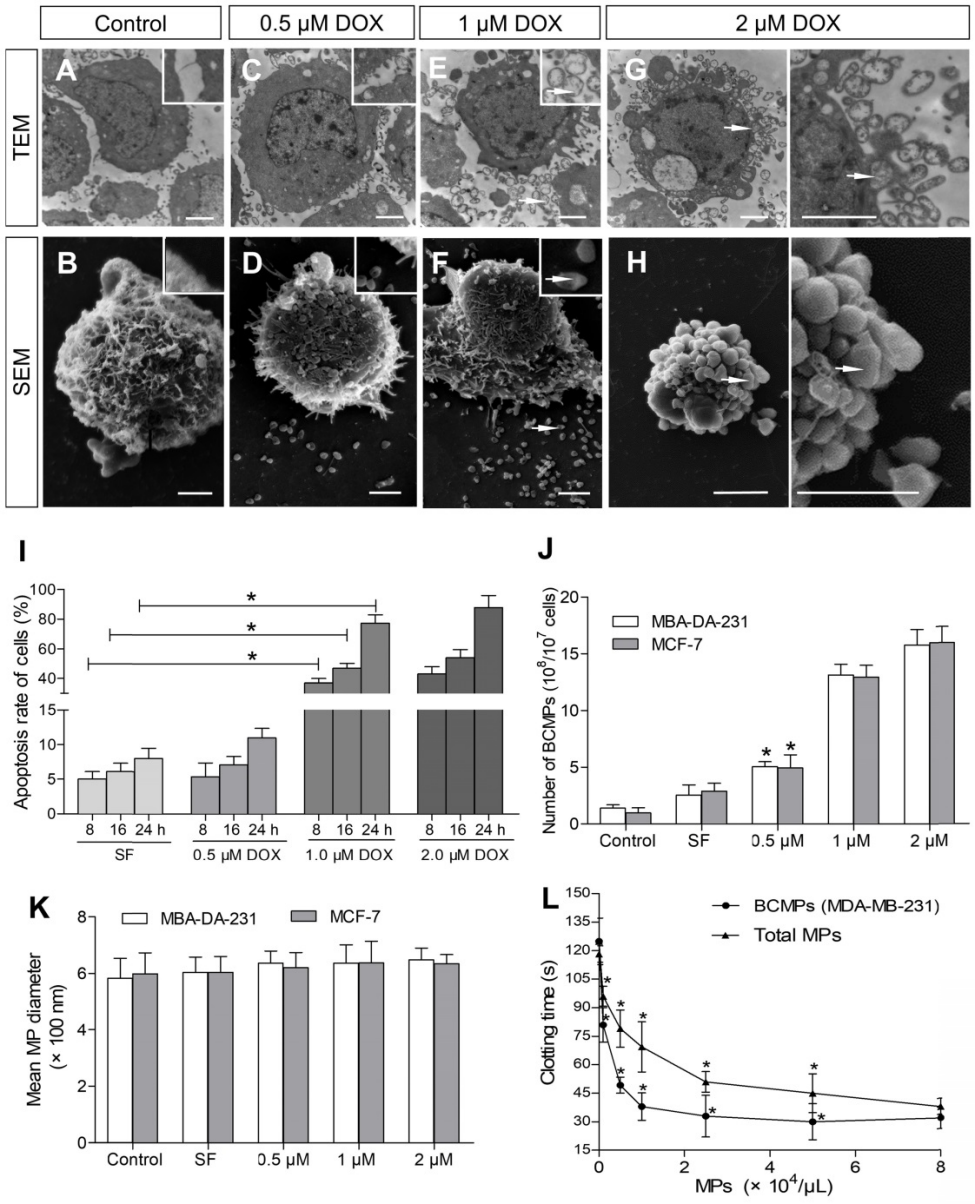
** Ultrastructure of DOX-treated MDA-MB-231 cells.** SEM and TEM images of MDA-MB-231 and MCF-7 cells incubated for 24 h with 0 (A, B), 0.5 (C, D), 1 (E, F), or 2 (G, H) μM of DOX. BCMPs are indicated by arrows. Scale bars represent 2 μm. (I) Apoptosis rate of MDA-MB-231 cells after DOX treatment. ^*^P < 0.001 vs. SF by two-way ANOVA. Number (J) and size (K) of isolated BCMPs. ^*^P < 0.001 vs. Control by two-way ANOVA. (L) Dose-dependent acceleration of plasma clotting time induced by total MPs and BCMPs (*n* =3). ^*^P < 0.001 vs. No MPs by repeated measures ANOVA. Abbreviations: BCMPs, breast cancer cell-derived microparticles; DOX, doxorubicin; MPs, microparticles; SEM, scanning electron microscopy; SF, serum-free medium; TEM, transmission electron microscopy.

TNBC MDA-MB-231 cells were incubated with various concentrations of DOX for 8-24 h to obtain BCMPs after *in vitro* NAC. Cells treated with SF were smooth with slender microvilli and intact intracytoplasmic structures and nuclei (Figure 2A-B). Similar treatment of MDA-MB-231 cells with 0.1 μM of DOX did not induce obvious apoptotic signals other than that present in control cells. In comparison, cells incubated with 0.5 μM of DOX exhibited fewer microvilli (Figure 2C-D). Cells treated with 1 μM and 2 μM of DOX were considerably smaller as well as rounder in shape compared to control cells, further suggesting apoptosis. SEM and TEM observation further revealed the possibility of BCMP expression around the cells after DOX treatment at 1 μM (Figure 2E-F, arrow) and 2 μM (Figure 2G-H, arrow). Thus, in the groups treated with the higher concentrations of DOX, apoptosis significantly increased (Figure 2I). Further, MDA-MB-231 cells undergoing apoptosis had well-defined cytoplasm, nuclear condensation, vacuolization of cytoplasm, and formation of considerable BCMPs. As many as 15.76 × 10^8^ BCMPs were yielded from 1 × 10^7^ MDA-MB-231 cells treated with 2 μM of DOX for 24 h. Besides MDA-MB-231 cells, the human breast cancer cell line MCF-7 also produced MPs after DOX treatment (data not shown), suggesting that cancer cells may indeed generate BCMPs in response to chemotherapy. We found that 2 μM of DOX caused >11.3-fold release of BCMPs from the tumor cell lines (Figure 2I) compared to the untreated group, as shown in Figure 2G. This observation further indicated that DOX can stimulate apoptotic signals in a dose-dependent manner along with increased formation of BCMPs (at 1 and 2 μM). The collected BCMPs were between 100 nm and 1 μm in diameter, which is different from exosomes and apoptotic bodies. There was no significant difference in their mean size between DOX-induced and SF-induced groups (Figure 2K, Figure S1). The BCMPs were found to shorten the clotting time of patient plasma in a dose-dependent manner (Figure 2L). At various *in vitro* concentrations (< 8 × 10^4^/μL), the pro-coagulant activity of isolated BCMPs was higher than that of circulating MPs in patients (Figure 2L).

### PS exposure on BCMPs supports formation of fibrin and facilitates assembly of prothrombinase

**Figure 3:**
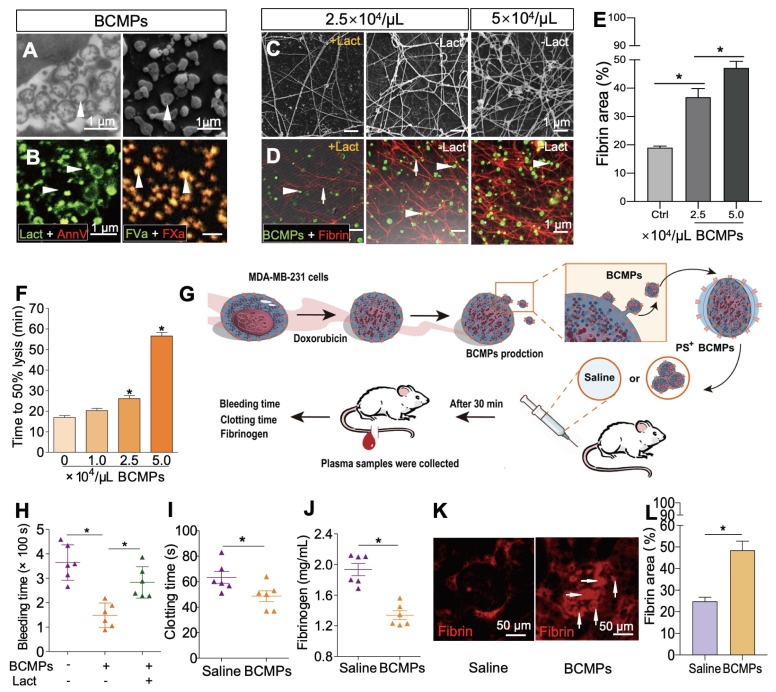
** PS^+^ BCMPs were procoagulant.** TEM (A, left) and SEM (A, right) images of BCMPs. (B, left) Confocal microscopy images of BCMPs co-stained with lactadherin (red) and annexin V (green) with colocalization indicated in yellow. (B, right) Confocal microscopy images of BCMPs co-stained for FVa (green) and FXa (red) with colocalization indicated in yellow. (C) SEM images of fibrin production by BCMPs in the presence of recalcified MP-depleted plasma with or without lactadherin. (D) Confocal microscopy images of fibrin production (red) by BCMPs (green). The BCMP clot contains BCMPs (arrowheads) on the surface of fibers (arrows) with increased branching points. (E) Fibrin area in the microscope field of view from D calculated using Image J. ^*^P < 0.05 by Student's *t*-test. (F) Time to attain 50% lysis of the formed fibrin clots, defined as the time elapsed from the maximal to half maximal absorption value at 405 nm (Lys 50_MA_). (G) Schematic illustration of an *in vivo* functional assay in which wildtype mice were injected with saline or 2.5 × 10^4^/μL BCMPs and then a tail vein bleeding test was performed. The total bleeding time (H), clotting time (I), and fibrinogen levels (J) were measured (*n* = 6; 3 independent experiments). ^*^P < 0.05 vs. Saline by Student's *t*-test. (K) Fluorescence microscopy images of dilated vessels in kidneys from BCMP-injected mice showing extensive fibrin deposition (arrowheads, right) compared with saline-injected mice (left). Images are representative of 6 mice in each group. (L) Fibrin area in the microscope field of view from K calculated using ImageJ. ^*^P < 0.05 by Student's *t*-test. Abbreviations: AnnV, annexin V; BCMPs, breast cancer cell-derived microparticles; Ctrl, control; DOX, doxorubicin; Lact, lactadherin; SF, serum-free medium.

The isolated BCMPs were not generated by background noise or nonspecific events and showed well-preserved membranes and intraluminal content (Figure 3A). Electron microscopy supported the expression of MPs with variable sizes by MDA-MB-231 cells, ranging from 100 to 1000 nm. Confocal analysis further confirmed exposure of PS on the surface of BCMPs by co-staining with lactadherin and annexin V (Figure 3B, left). Fluorescence labeling of FVa and FXa on BCMPs was performed in order to assess whether PS exposure directly correlated with local PCA by facilitating prothrombinase assembly. It can be concluded that MPs offer the best biological surface for binding, which was evident through colocalization of bound FVa and FXa on BCMPs (Figure 3B, right). This surface was most likely achieved through externalization of PS. We further sought to explore whether BCMPs promote the formation of fibrin clots in a similar manner to that of cellular PCA. This was determined by incubating BCMPs with normal recalcified MP-depleted plasma and evaluating fibrin production by SEM (Figure 3C) and anti-fibrin staining (Figure 3D). After analysis, a large amount of fibrin was observed to be spread around the BCMPs (Figure 3E). The higher the concentration of BCMPs, the higher the average fibrin density. Further, the density of the fibrin network was found to be higher in areas near the BCMPs, which indirectly proposes the formation of fibrin through exposed PS. Based on these changes in fibrin structure, we evaluated the impact of BCMPs on tPA-induced lysis of fibrin made from purified fibrinogen in the presence of plasminogen. The time to attain 50% lysis of the fibrin clots was prolonged in the presence of increasing concentrations of BCMPs (Figure 3F). In addition, incubation of BCMPs with lactadherin or TF antibody lowered the formation of fibrin to different levels (Figure S2), suggesting that BCMPs can induce high PCA, which can be reduced by TF antibody and markedly inhibited by lactadherin. Combined with these *in vitro* findings, isolated BCMPs injected into mice (Figure 3G) induced a hypercoagulable state within 30 min, characterized by shortened bleeding (Figure 3H) and clotting (Figure 3I) times, decrease level of plasma fibrinogen (Figure 3J), and increased fibrin deposition in the kidney vasculature (Figure 3K-L). Lactadherin reversed these changes and the hypercoagulable state (Figure 3H).

### Effects of BCMPs on platelets

The expression of PS on BCMPs may explain why PCA was enhanced by MPs. However, it left unexplained why relatively few BCMPs could induce considerable thrombotic trends, as shown in Figure 2L. One possibility is that blood cells stimulated by BCMPs convert to a new phenotype that is also procoagulant. To test this hypothesis, *in vitro* assays were conducted to study the effect of BCMPs on the PCA of platelets.

**Figure 4:**
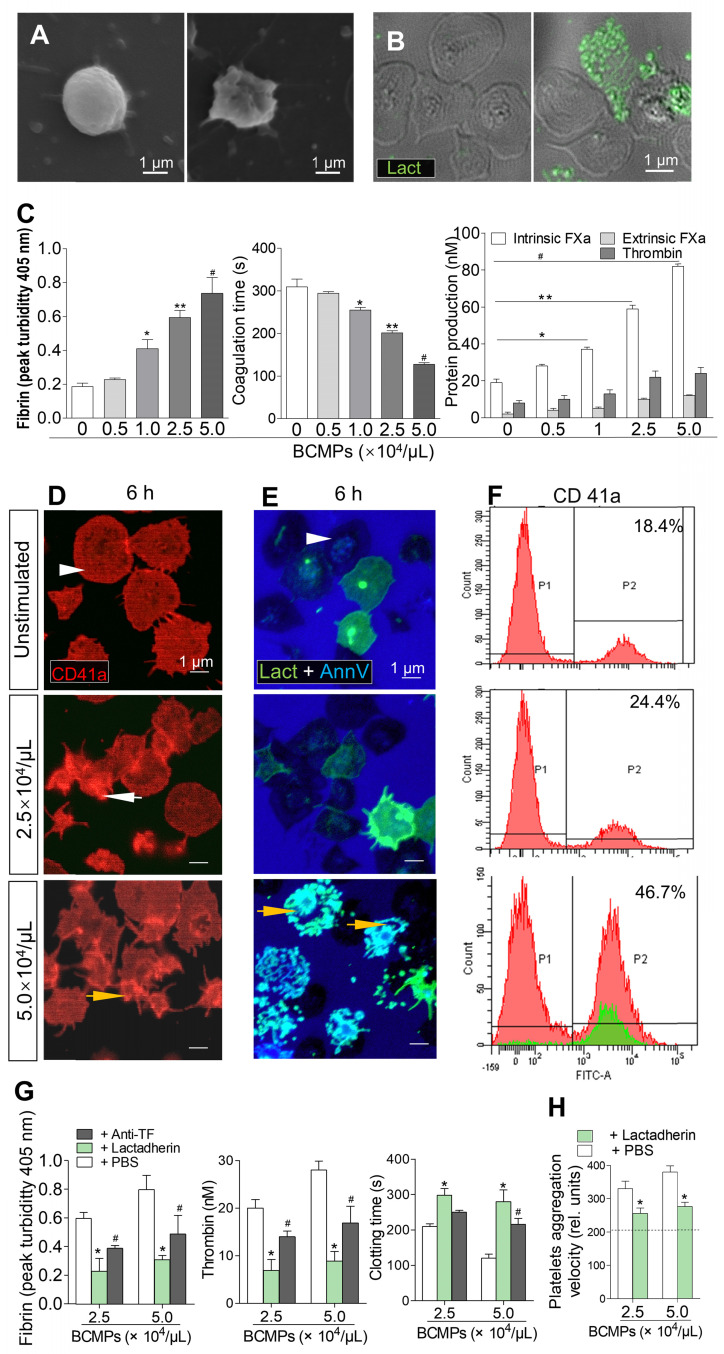
** Effect of BCMPs on platelets.** (A) SEM images of quiescent (left) and activated (right) platelets. (B) Confocal microscopy images of quiescent (left) and apoptotic (right) platelets stained with lactadherin (green). (C) Fibrin formation, coagulation time, and protein production of intrinsic/extrinsic FXa and thrombin by platelets treated with BCMPs (*n* = 3). ^*^P < 0.05, ^**^P < 0.01, ^#^*P* < 0.001 vs. No BCMPs by Student's *t*-test. (D) Confocal microscopy images of CD41a exposure (red) on platelets incubated with MDA-MB-231 BCMPs for 6 h. (E) Confocal microscopy images of platelets incubated with MDA-MB-231 BCMPs for 6 h and stained with lactadherin (green) and annexin V (blue). Quiescent platelets (white arrowhead), activated platelets (white arrow), and apoptotic platelets (yellow arrow) are marked. (F) Flow cytometry results of washed platelets (1 × 10^8^/mL) incubated with BCMPs for 6 h. Platelet-derived MPs are indicated in the green histogram trace. (G) Fibrin formation, thrombin level, and clotting time of platelets incubated with BCMPs and treated with lactadherin or TF antibodies (*n* = 3). ^*^P < 0.01, ^#^*P* < 0.05 vs. PBS by Student's *t*-test. (H) Aggregation velocity of platelets incubated with BCMPs and treated with lactadherin. Data from the control group are represented by the dotted line (*n* = 3). ^*^P < 0.01 vs. PBS by Student's *t*-test. Abbreviations: AnnV, annexin V; BCMPs, breast cancer cell-derived microparticles; FITC, fluorescein isothiocyanate; FXa, factor Xase; Lact, lactadherin; PBS, phosphate-buffered saline.

Platelets isolated from healthy subjects were treated with various concentrations of BCMPs. In the control samples, the round platelet shape was retained (Figure 4A, left). After treatment with BCMPs, the number of platelet MPs increased in comparison to the untreated control group, further indicating that BCMPs are responsible for inducing activation of platelets and associated apoptosis along with higher PCA. SEM further revealed that the platelets were stimulated by high-dose treatment of BCMPs, with pseudopodia extensions and apoptosis being observed (Figure 4A, right). These results were consistent with those obtained through confocal microscopy (Figure 4B). At concentrations of BCMPs greater than 1.0 × 10^4^/μL (Figure 4C, right) there was a significant increase in the formation of intrinsic FXa, which further promoted the formation of BCMPs at greater than 2.5 × 10^4^/μL. Although a very small increase in extrinsic FXa was observed upon platelet treatment with BCMPs, PCA was found to increase in a dose-dependent manner, resulting in increased fibrin (Figure 4C, left) and decreased coagulation time (Figure 4C, middle).

Platelets isolated from healthy subjects and patients were stimulated with high (5.0 × 10^4^/μL) and low (2.5 × 10^4^/μL) concentrations of BCMPs for 6 h. Structural analysis of the platelets by confocal microscopy revealed that they underwent morphological changes and had become fragmented as well as crenulated (Figure 4D-E, arrows). Platelets in the high dose groups showed greater exposure of PS and formed extracellular vesicles (Figure 4E, bottom). The platelet MPs were radially scattered around activated platelets (Figure 4E, yellow arrow). Similarly, flow cytometry analysis showed that CD41a expression was greatly increased in the high dose groups (46.7%) compared to the low dose groups (24.4%) (Figure 4F).

### Effects of TF antibody and lactadherin on PCA

We next determined the blocking effect of TF and PCA on platelets after BCMPs treatment. Platelets were incubated with 2.5 × 10^4^ or 5.0 × 10^4^/μL of BCMPs for 1 h, treated with TF antibody, lactadherin, or PBS, and then coagulation time, extent of thrombin production, and fibrin generation were measured. Treatment with TF antibody and lactadherin reduced the production of fibrin (Figure 4G, left) as well as thrombin (Figure 4G, middle) and prolonged coagulation time (Figure 4G, right). Because it also blocks PS, lactadherin inhibited platelet PCA by more than 60%. Therefore, while the TF antibody alleviated BCMP-induced PCA, lactadherin caused greater inhibition (Figure 4G). The enhanced PCA was related to increased fibrin and decreased coagulation time and platelet aggregation velocity (Figure 4H), which were all reversed by lactadherin (Figure 4G-H).

### BCMPs trigger domino-like PS exposure on HUVECs

**Figure 5:**
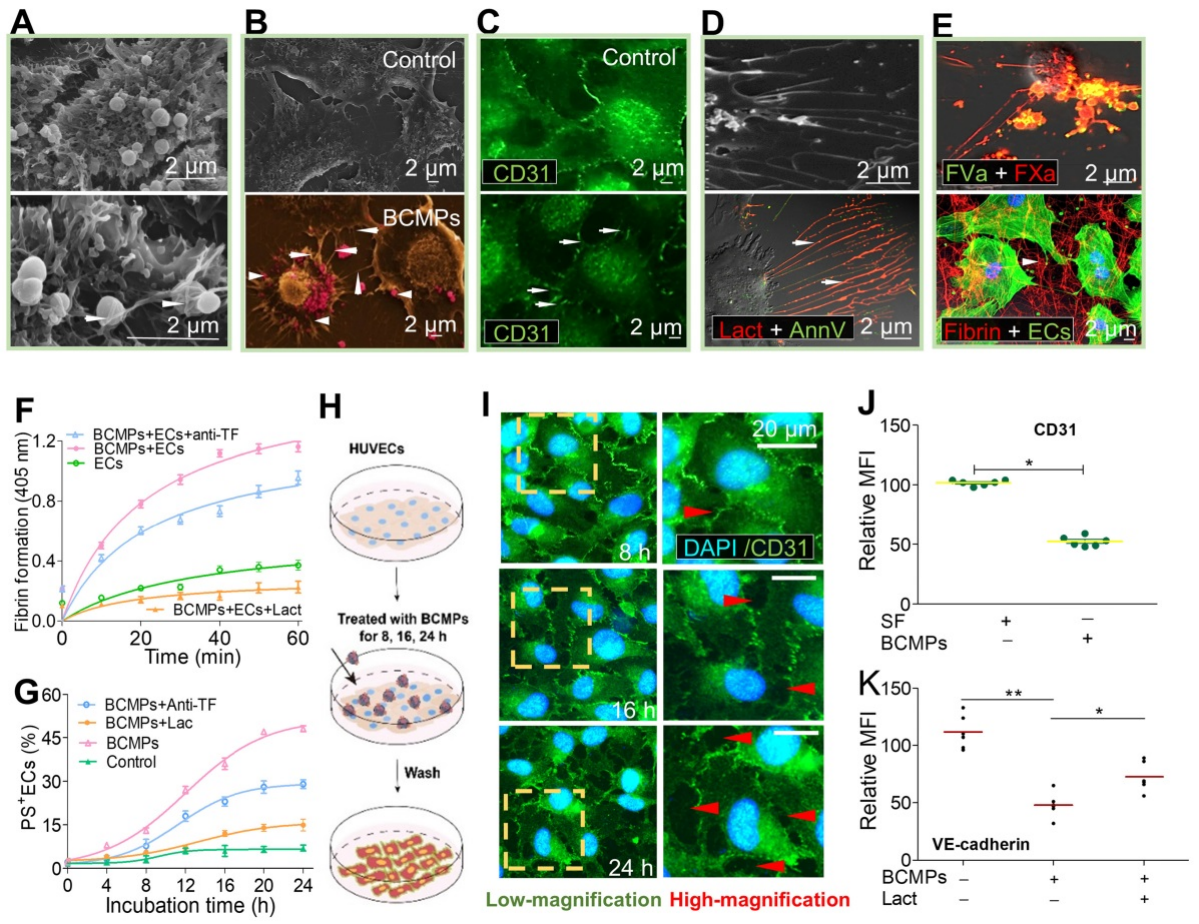
**BCMPs converted ECs to a procoagulant phenotype and compromised endothelial monolayer integrity.** HUVECs were incubate with or without BCMPs (5.0 × 10^4^/μL) for 24 h. (A) SEM images of grasped (bottom, left arrow) and internalized (bottom, right arrow) target BCMPs by HUVECs. (B) SEM images showing the morphology of HUVECs incubated without (top) or with (bottom) BCMPs. BCMPs with a spherical structure were pseudocolored pink. (C) Confocal microscopy images of HUVECs stained with Alexa Fluor 488-conjugated CD31 antibody. (D, top) SEM image of HUVECs with extending pseudopodia. (D, bottom) Confocal microcopy image of PS exposure on HUVEC filopods (arrows) by annexin V (red) and lactadherin (green) co-staining. (E, top) Confocal microscopy images of colocalized (yellow) FVa (green) and FXa (red) on filopods near the retracted margins of HUVECs and on newly formed thin filaments. (E, bottom) Confocal microscopy images of HUVECs pretreated with DOX-induced BCMPs and incubated with healthy plasma showing considerable fibrin strand formation arranged radially along the filopodia to create a fibrin network (arrowhead). (F) Fibrin production on BCMP-cultured ECs in the presence of recalcified MP-depleted plasma (*n* = 3). (G) Kinetics of PS exposure on HUVECs in response to BCMPs with or without lactadherin (128 nM) or TF antibody (25.6 μg/mL) (*n* = 3). (H) Schematic illustration of the co-incubation assay. (I) Confocal microscopy images of HUVEC monolayer stained with DAPI (blue) and CD31 (green) following treatment with BCMPs for 8, 16, or 24 h to induce leakiness (red arrowheads). (J) Relative expression of CD31 on HUVECs stimulated with BCMPs for 24 h measured by immunofluorescence imaging (*n* = 3). Expression is indicated by mean fluorescence intensity (MFI). ^*^P < 0.05 by Student's *t*-test. (K) VE-cadherin expression on HUVECs stimulated with BCMPs for 24 h and treated with lactadherin measured by flow cytometry (*n* = 3). ^*^P < 0.05, ^**^P < 0.01 by paired Student's *t*-test. Abbreviations: AnnV, annexin V; anti-TF, tissue factor antibody; BCMPs, breast cancer cell-derived microparticles; ECs, endothelial cells; Lact, lactadherin.

To analyze the effect of BCMPs on EC viability and apoptosis, a high concentration of BCMPs (5.0 × 10^4^/μL) was added to cultured HUVECs for 24 h. The BCMPs were trapped (Figure 5A, bottom left, arrow) and internalized (Figure 5A, bottom right, arrow) through the extension of pseudopodia from HUVECs. We further observed that PS exposure on HUVECs was increased by BCMP induction. SEM pinpointed effective structural changes such as retraction of cellular edges as well as increased generation of filopodia (arrows) after stimulation by BCMPs (arrowheads) (Figure 5B). To improve contrast, the BCMPs were pseudocolored pink (Figure 5B, Bottom). Results from confocal microscopy were similar (Figure 5C). To detect PS expressed on the pseudopodia, the HUVECs were co-stained with Alexa Fluor 488-annexin V and Alexa Fluor 647-lactadherin. PS exposure was apparent on the filopodia as well as some localized areas of the HUVECs (Figure 5D). ECs activated by BCMPs may provide a supplementary procoagulant surface due to PS exposure. HUVECs incubated with BCMPs (5.0 × 10^4^/μL) showed increased production of thrombin and other complexes like extrinsic and intrinsic FXa in comparison to cells treated with SF, confirming this hypothesis. In addition, integrated PCA was evaluated in HUVECs using a fibrin generation assay and by coagulation time. A significant fraction of bound FXa and FVa were colocalized, as observed by confocal microscopy (Figure 5E, upper). This result indicates that cells cultured with BCMPs offer biological support to facilitate binding of coagulation factors, most evidently through externalized PS. As expected, HUVECs treated with BCMPs formed massive amounts of fibrin (Figure 5E, lower), which further enhanced clotting. The contributions of TF and PS were further assessed using a fibrin generation assay on BCMP-treated HUVECs preincubated with TF antibody and lactadherin. Interestingly, fibrin generation (Figure 5F, Figure S3) and PS^+^ ECs (Figure 5G) were found to be decreased slightly by TF antibody but significantly by lactadherin.

### BCMPs compromise endothelial monolayer integrity and increase transendothelial migration

BCMPs (2.5 × 10^4^ or 5.0 × 10^4^/μL) were added to cultured ECs for various incubation times. Morphological changes to the ECs and rupture of tight junctions occurred simultaneously with PS exposure. HUVECs incubated with 2.5 × 10^4^ BCMPs for 16 or 24 h had altered morphology and retracted from cell-cell junctions, but showed minimal changes at 8 h (Figure 5H-I). Consistent with this result, long incubation times triggered a marked increase in the retraction of cell margins compared with short incubation times (Figure 5I, red arrowheads). Additionally, both CD31 and VE-cadherin expression levels were significantly decreased on BCMP-treated HUVECs (Figure 5J-K). Pretreatment of PS^+^ BCMPs with lactadherin reduced the BCMP-induced loss of VE-cadherin expression (Figure 5K) and transendothelial gaps compared to untreated groups.

**Figure 6:**
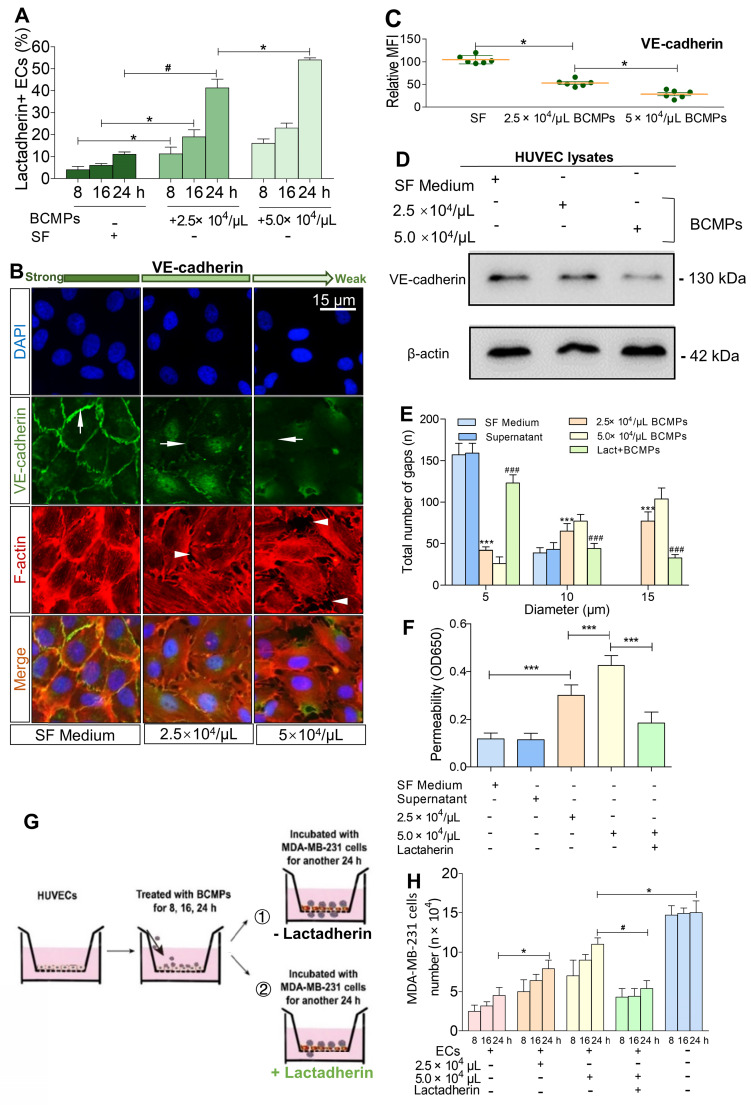
** Breast cancer cells exploited the increased endothelial permeability induced by BCMPs.** (A) Proportion of lactadherin-positive ECs in HUVECs incubated with BCMPs (*n* = 3). ^*^P < 0.05, ^#^*P* < 0.01 by Student's *t*-test. (B) Immunofluorescence images of HUVEC monolayers incubated with BCMPs for 24 h. The cells were stained with DAPI (blue) and for VE-cadherin (green) and F-actin (red). Arrows point to leaky HUVECs. (C) Relative expression of VE-cadherin on HUVECs incubated with BCMPs for 24 h measured by immunofluorescence imaging. Expression is indicated by mean fluorescence intensity (MFI). ^*^P < 0.05 by Student's *t*-test. (D) Western blots of VE-cadherin expression in HUVECs incubated with BCMPs. (E) Size distribution of intercellular gaps in HUVEC monolayers stimulated with various treatments (*n* = 200 gaps from 10 glass coverslips). ^***^P < 0.001 vs. SF, ^###^*P* < 0.001 vs. 5.0 × 10^4^/μL BCMPs by two-way ANOVA. (F) Permeability of HUVEC monolayers stimulated with various treatments for 24 h (*n* = 6). Lact + BCMPs: lactadherin + 5.0 × 10^4^/μL BCMPs. ^***^P < 0.001 by Student's *t*-test. (G) Schematic illustration of the migration assay. Monolayer HUVECs were stimulated with SF or BCMPs and then incubated with MDA-MB-231 cells in the presence or absence of lactadherin. (H) Number of migrated MDA-MB-231 cells. ^*^P < 0.05, ^#^*P* < 0.01 by Student's *t*-test. Abbreviations: BCMPs, breast cancer cell-derived microparticles; ECs, epithelial cells; OD, optical density; SF, serum-free medium; Lact, lactadherin.

We found that treatment of HUVECs with BCMPs increased PS^+^ HUVECs in a dose- and time-dependent manner (Figure 6A). To further characterize endothelial integrity, immunofluorescence staining of VE-cadherin and F-actin was performed (Figure 6B). We found that the effects of BCMPs on HUVEC permeability may be due to transient disruption of VE-cadherin-based cell-cell adhesion. In the control group, stable junctions were marked by faint cortex F-actin, which were aligned by thick parallel actin bundles that did not overlap with VE-cadherin. In contrast, BCMP-treated (2.5 × 10^4^ and 5.0 × 10^4^/μL) HUVECs showed structural aberrations such as uncontrolled actin polymerization and subsequent disassembly of VE-cadherin (Figure 6C-D). BCMPs moderately increased the number of intercellular gaps, which was most prominent in the higher dose group (5.0 × 10^4^/μL; Figure 6B, E). These data led us to believe that BCMPs increased endothelial junction disruption and raised cytoskeletal tension, which promoted vascular leakage in a dose- and time-dependent manner. Such leakiness in the endothelium may allow large numbers of BCMPs and a few circulating tumor cells to break into the blood stream, causing platelet activation, systemic vascular endothelium disruption, and potentially even distant metastasis. Interestingly, only the isolated SF-induced and DOX-induced BCMPs, but not the supernatants from the last wash, were cytotoxic (Figure 6E-F) and procoagulant (Figure S4, Figure S5), suggesting that the cytotoxic/procoagulant substance was within or adhered to the BCMP membrane and was not discharged from the BCMPs. This result is in line with the previous result in Figure 3A, which showed that the BCMPs were relatively stable.

### Lactadherin reverses BCMP-mediated prometastatic changes

**Figure 7:**
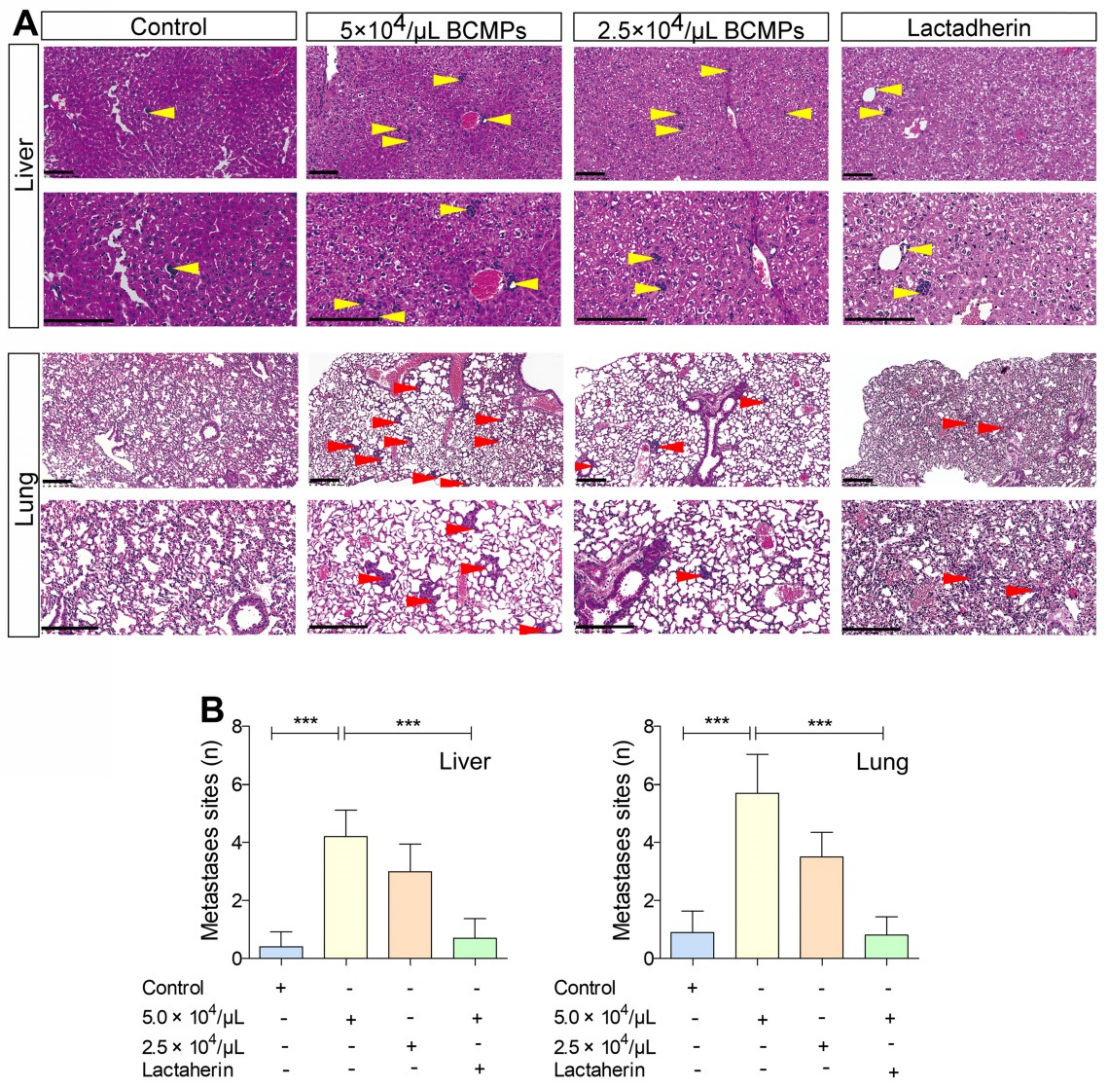
** BCMPs increased metastasized tumor burden in the liver and lungs in a dose-dependent manner.** (A) H&E-stained histological images of liver and lung tissues from mice treated with BCMPs or lactadherin and injected with MDA-MB-231 cells. Tumors are indicated by yellow and red arrowheads. The images are representative of three mice. Scale bars represent 200 μm. (B) Quantification of metastases in the liver and lung. ^***^P < 0.001 by Student's *t*-test. Abbreviations: BCMPs, breast cancer cell-derived microparticles.

We investigated if BCMPs have a role in enhancing the migration of cancer cells across the endothelial barrier. Treatment of HUVECs with BCMPs resulted in a concentration-dependent enhancement in permeability (Figure 6F). We next performed a transwell permeability assay with HUVECs and MDA-MB-231 cells (Figure 6G). When BCMP-treated HUVECs were co-incubated with lactadherin, the extent of cancer cell migration was reduced (Figure 6H, green histograms). *In vivo*, even though both the BCMP-treated group and the control group received intravenous MDA-MB-231 cells, only the former had multiple metastases in the liver (Figure 7A, upper). There were more lung metastases in the BCMP-treated group than in the control group (Figure 7A, lower), especially in the group treated with the higher concentration of BCMPs (Figure 7B). Consistent with the *in vitro* results, the lactadherin-treated group had fewer metastases. All these *in vitro* and *in vivo* results indicate that BCMPs induced leaky vasculature that led to easier extravasation of cancer cells.

**Figure 8:**
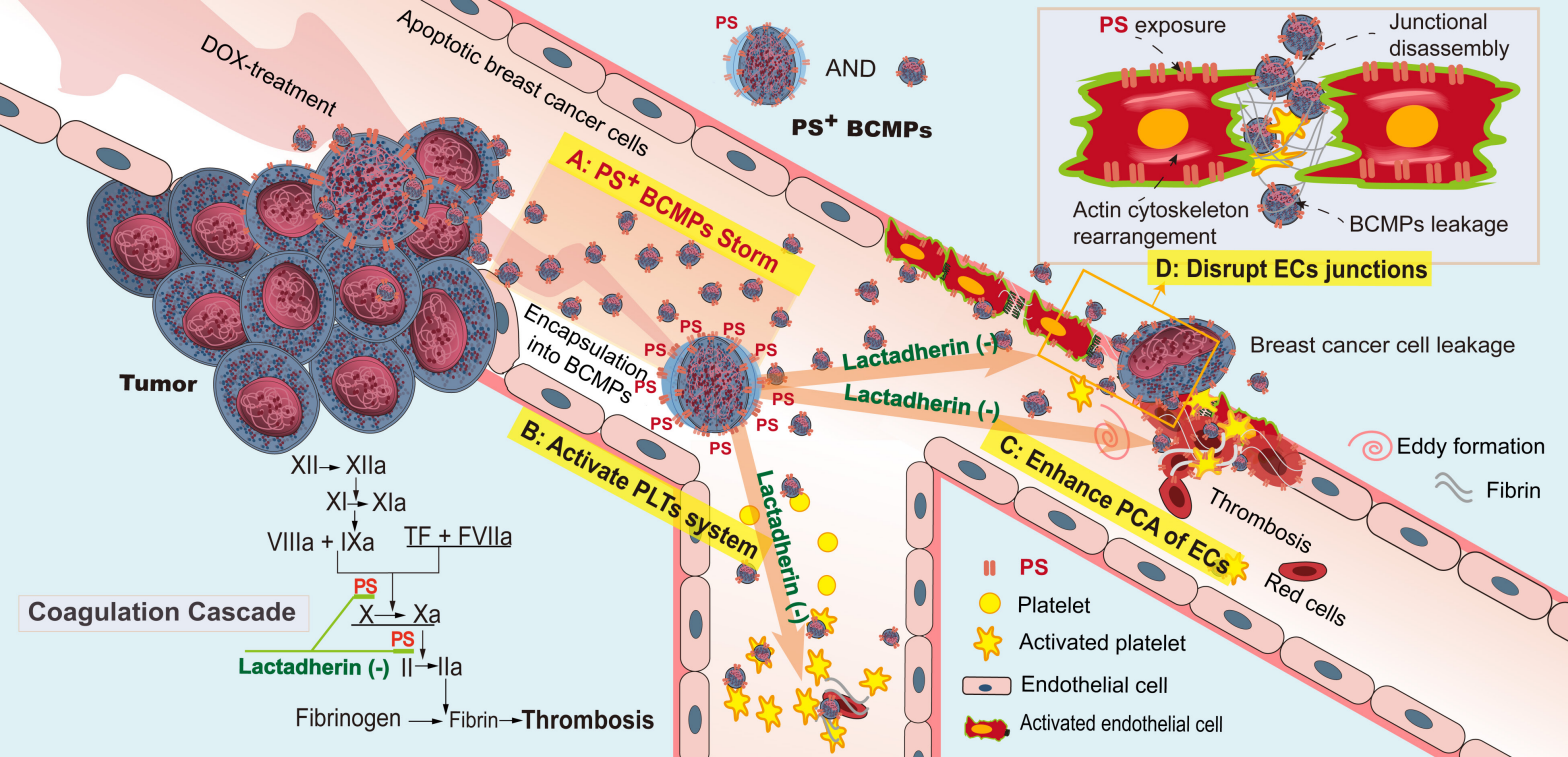
** PS^+^ BCMPs exacerbated coagulation and cancer cell transendothelial migration in TNBC.** Chemotherapy induced a massive release of PS^+^ BCMPs. PS exposure on BCMPs supported the formation of fibrin and facilitated the assembly of prothrombinase. At the same time, PS^+^ BCMPs activated the platelet system (B) and enhanced the procoagulant activity of ECs (C) to further build a hypercoagulable state. In addition, BCMPs destroyed EC junctions (D) and enabled distant cancer cell metastasis. However, lactadherin reversed these hypercoagulable and high metastatic states. Abbreviations: BCMPs, breast cancer cell-derived microparticles; DOX, doxorubicin; ECs, endothelial cells; PLTs, platelets; PS, phosphatidylserine; TF, tissue factor.

Taken together, the above clinical, *ex vivo*, *in vitro*, and *in vivo* findings support the role of PS^+^ BCMPs in TNBC after NAC through stimulating activation of a local coagulation cascade and facilitating metastatic colonization of circulating cancer cells (Figure 8). Lactadherin diminished PCA and increased the rate of cancer cell clearance.

## Discussion

We made four significant observations. Firstly, increased PS^+^ BCMPs was correlated with increased plasma PCA. Moreover, PCA was significantly inhibited by lactadherin, while the effect of TF antibody was minimal. Secondly, confocal microscopy showed PS exposure and colocalized deposition of FVa and FXa on isolated BCMPs. PS^+^ BCMPs activated platelets and induced scattered apoptosis, further facilitating high PCA. Thirdly, the isolated BCMPs exerted a strong effect on EC activation, further transforming them to a procoagulant phenotype. And lastly, PS^+^ BCMP-induced EC leakiness enabled cancer cell migration, while lactadherin reversed BCMP-mediated prometastatic changes. Thus, the collective *in vivo* evidence together with other cellular events clarified that PS^+^ BCMPs worsened the hypercoagulability state and cancer metastasis in a dose- and time-dependent manner. All these findings make PS^+^ BCMPs an attractive therapeutic target for the prevention of cancer-associated abnormal hypercoagulability and metastasis.

Notably, 50% of patients treated with NAC still have residual tumor and comparatively poor outcomes such as formation of acute lower extremity deep venous thrombosis or tumor recurrence 50-55. Underglycosylated MUC1 is overexpressed 56, 57 and present on MPs of epithelial breast cells in the majority of breast cancers 25, 40, 58. There has been a degree of uncertainty and debate over whether BCMPs are capable of reaching adequate concentrations within the plasma milieu to elicit these profound effects on hemostasis. Our data revealed a prominent increase in the number of MUC1^+^ MPs 1 day after NAC, suggesting that the body may not be able to clear a large number of BCMPs efficiently. It is reasonable to assume that variations in their rate of generation or impairment of the mechanism of clearance may lead to accumulation of circulating procoagulant BCMPs, which would enhance or prolong their thrombotic effect in patients treated with NAC.

The negative surface charge of the external leaflet of the MP membrane determines the targeting of proteins containing polycationic motifs, such as coagulation factors. Previous studies, including our own 23, 33, suggest that the unique negativity of the MP external leaflet is attributed, in part, to its high PS content. We used the PS probe lactadherin to study the distribution of PS. We previously found that lactadherin bound specifically to PS with a sensitivity 10-100 times higher than that of annexin V 33. Here, we further showed that DOX-induced BCMPs had increased PCA associated with PS exposure, which was blocked by lactadherin. We demonstrated *ex vivo* that PS^+^ BCMPs stimulated activation of platelets and apoptosis, facilitating the formation of thrombin, fibrin, and intrinsic FXa. In the coagulation cascade, the intrinsic and extrinsic pathways converge at FX activation, where one molecule of FXa results in the generation of 1000 molecules of thrombin 59. FXa combined with FVa converts prothrombin into thrombin, which cleaves fibrinogen to form fibrin. Thus, increased FXa contributes to increased fibrin, leading to shortened coagulation times. Binding alters the structure of the fibrin clot, altering its susceptibility to degradation by fibrinolytic enzymes. In the present study, BCMP-treated platelets had significantly elevated PS exposure. Further blockade of platelet PS with lactadherin inhibited the production of thrombin and fibrin by ~60%. TF had relatively little effect on the PCA of BCMPs. In conclusion, accumulated PS^+^ BCMPs after NAC may stimulate activation and apoptosis of platelets, further contributing to the hypercoagulative state and complications associated with thrombosis in TNBC.

Virchow's triad, which explains the underlying physiological mechanisms in the pathogenesis of thrombosis, pinpoints concerted roles of platelet abnormalities, endothelium abnormalities, and restriction of blood flow in the development of arterial as well as venous thrombosis 40, 59. Consistent with this understanding, co-incubation of HUVECs with BCMPs to mimic the *in vivo* conditions revealed significant dose- and time-dependent PS exposure on HUVECs, cytoskeletal tension, and EC leakiness. Compared with the ability of BCMPs or HUVECs to produce fibrin alone, HUVECs treated with BCMPs have the highest ability of fibrin formation. In addition, the distribution of PS^+^ BCMPs by confocal microscopy was irregular, punctate, and intermittent, while the distribution of PS on stimulated ECs was regular and continuous. These results indicate that BCMPs activated the PCA of HUVECs, rather than simply adhesion. More importantly, we demonstrated that HUVECs treated with PS^+^ BCMPs had co-bound FVa and FXa on their filopodia. PS acts as an anchor for the assembly of the prothrombinase complex, which converts fibrinogen to fibrin. Furthermore, the consistent PS and fibrin distribution suggests that HUVECs contributed to local coagulation directly via PS exposure. Thus, the results indicate that HUVECs were converted to a procoagulant phenotype and facilitated thrombosis.

Transendothelial migration of cancer cells is central to the pathophysiology of metastasis. Even if the hepatic blood vessel is fenestrated (~20-250 nm wide), it may not be wide enough for cancer cell migration unless the endothelial barrier is damaged by BCMPs. The integrity of the EC monolayer is challenged by PS^+^ BCMPs due to degradation of VE-cadherin-based intracellular junctions and jumbling of the actin cytoskeleton 60. Deactivation or genetic deletion of the adhesive function of VE-cadherin leads to enhanced vessel permeability 61, whereas increasing VE-cadherin-dependent adhesion protects the integrity of the endothelial barrier 62. An impaired endothelium may present less of a challenge to a metastatic tumor cell trying to pass through the vessel wall. Therefore, BCMPs may facilitate entry of metastatic cells to previously inaccessible tissue sites. Our evidence suggests that BCMPs assist cancer cells in exiting circulation and create opportunities for cancer cell proliferation within sites of metastasis. Thus, BCMP-mediated EC dysfunction and/or morphology change facilitate both the hypercoagulable state and cancer cell transendothelial migration in malignant disease.

Nearly all MPs (whether pathological or originating from activated healthy cells) display high PS exposure. However, PS^+^ BCMPs are released in large quantities only under pathological conditions or following intervention with external stimuli. In the condition of NAC, if the body cannot instantly eliminate the massive amounts of formed PS^+^ BCMPs, they will induce a domino-like effect. We prefer to define this situation as a PS^+^ BCMPs Storm because accumulated PS^+^ BCMPs result in a hypercoagulable state and a high extravasation state, which increase the risk of metastasis and death. In future treatment strategies, two steps should be considered: effective killing of tumors followed by blocking of PS^+^ BCMPs. De et al. reported a novel PS-targeting liposome bearing phosphatidylcholine-stearylamine and entrapping DOX that induced apoptosis and showed potent anticancer effects as a single agent against a majority of cancer cell lines 41, 42. Similar strategies might both kill the tumor and directly mitigate MP release with a single treatment.

This study has some limitations. Quantitation of PS^+^ BCMPs by flow cytometry is prone to variation; thus, preparation and analysis of clinical samples need to be standardized. In addition, the role of BCMPs is complex and pleiotropic; therefore, further study is needed to ascertain the molecular mechanisms underlying BCMP effects.

## Conclusions

Interactions between BCMPs and biological systems may need to be considered when assessing cancer development with NAC. BCMPs can accumulate over long durations of chemotherapy, inducing a hypercoagulability state and worsening opportunistic metastases in new sites despite cessation of chemotherapy. Given the well-delineated side effects of BCMP-induced endothelial leakiness and hypercoagulability, an in-depth understanding is required to improve control of these negative phenomena. From another perspective, exploitation of BCMP-related endothelial leakiness may improve the delivery of liposomes to tumors. Lactadherin, a PS-binding scavenging molecule, may improve the clinical benefits of NAC. Our discovery of the mechanism underlying BCMP-driven hypercoagulability and enhanced cancer cell transendothelial migration can be exploited to develop efficient drug delivery approaches for a variety of cancer pathologies including, but not limited to, breast malignancies and cancer-associated thrombosis.

## Supplementary Material

Supplementary figures.

## Figures and Tables

**Table 1:** Characteristics of healthy control individuals and patients with TNBC undergoing NAC.

	Control (*n* = 20)	TNBC (*n* = 18)			
		Day 0	Day 1	Day 2	Day 6
Age (y)	53.1±8.3	50.1±10.8	-	-	-
BMI (kg/m^2^)	28.2±3.9	29.3±5.1	-	-	-
Smoking, n (%)	6 (30)	9 (36)	-	-	-
Dyslipidemia, n (%)	2 (10)	6 (24)	-	-	-
Hypertension, n (%)	2 (10)	5 (20)	-	-	-
PT (s)	11.80±1.83	12.10±1.13	12.88±1.42	12.43±1.21	12.10±1.32
aPTT (s)	22.50±3.42	27.50 ±3.22^**^	28.49±3.06	29.29±2.19	27.23±2.41
TT (s)	18.80± 0.16	20.22±0.31^*^	22.31±1.29^##^	21.31±1.98	19.12±0.46
D-dimer (ng/mL)	283±14	629±45^**^	738±42^##^	793±32	608±49
Fibrinogen (g/L)	2.65±0.75	4.9±0.45^**^	5.69±0.63^#^	6.29±0.49	4.8±0.56

Abbreviations: BMI: body mass index; PT, prothrombin time; aPTT, activated partial thromboplastin time; TT, thrombin time; TNBC, triple-negative breast cancer. ^*^P < 0.001, ^**^P < 0.0001 vs. Control; ^#^*P* < 0.001, ^##^*P* < 0.0001 vs. Day 0.

**Table 2:** Flow cytometry analysis of circulating MPs in healthy control individuals and patients with TNBC undergoing NAC.

Origin of lactadherin^+^ MPs (/μL)	Control	Breast cancer			
		Day 0	Day 1	Day 2	Day 6
Lactadherin^+^ MPs	2061 ± 124	2861 ± 237^*^	3630 ± 632^#^	4332 ± 654	2960 ± 206
Breast cancer cells (MUC1)	-	54 ± 10^*^	430 ± 111^#^	830 ± 125	330 ± 70^#^
Platelets (CD41a^+^)	1035 ± 195	1528 ± 225^*^	2073 ± 422^#^	2243 ± 391	1728 ± 205
ECs (CD31^+^ CD41a^-^)	89 ± 24	219 ± 46^*^	303 ± 72^#^	333 ± 52	300 ± 41^#^
TF^+^ MPs (CD142^+^)	18 ± 4	23 ± 5^*^	104 ± 3^#^	146 ± 4	26 ± 3

Abbreviations: ECs, endothelial cells; MPs, microparticles; TF, tissue factor. ^#^*P* < 0.0001 vs. Day 0; ^*^P < 0.0001 vs. Control.

## Abbreviations

aPTT: activated partial thromboplastin time
BCMPs: breast cancer cell-derived microparticles
BSA: bovine serum albumin
DOX: doxorubicin
ECs: endothelial cells
EDTA: ethylenediaminetetraacetic acid
FVa: factor Va
FXa: factor Xase
HUVECs: human umbilical vein endothelial cells
MPs: microparticles
MUC1: mucin 1
NAC: neoadjuvant chemotherapy
PBS: phosphate-buffered saline
PCA: procoagulant activity
PFP: platelet-free plasma
PS: phosphatidylserine
PT: prothrombin time
SEM: scanning electron microscopy
SF: serum-free medium
SWE: shear wave elastography
TAT: thrombin-antithrombin complex
TEM: transmission electron microscopy
TF: tissue factor
TNBC: triple-negative breast cancer
TRITC: tetramethylrhodamine
tPA: plasminogen activator
TT: thrombin time
VTE: venous thromboembolism
