# Supplementary material for: Phosphatidylserine-exposing tumor-derived microparticles exacerbate coagulation and cancer cell transendothelial migration in triple-negative breast cancer
Source: Theranostics. 2021 Apr 19;11(13):6445–60. doi: 10.7150/thno.53637 (PMC8120203; doi:10.7150/thno.53637)
Supplement: Supplementary file 1 — Supplementary figures. [file thnov11p6445s1.pdf]

Supplementary Methods

Flow cytometry analysis

Both the PS and TF expression were quantified with the help of flow analysis. Around 1×10^6 platelets per mL were incubated with the corresponding fluorochrome tagged antibody, in this case, Alexa 647-anti-CD142 (5 nM) and Alexa 488-labeled lactadherin (2 nM) for about 15 min at room temperature, in dark. Around 5 µL of suspension rich with MPs was diluted using 35 µL of Tyrod's buffer and stained individually with Alexa 488-anti-MUC-1 (5 nM) and Alexa 647-anti-CD41a (5 nM) at 4 °C for 15 min in dark. MPs derived from platelet-rich plasma were identified using CD41a. Whereas BCMPs were tagged with MUC-1. The BCMPs number was obtained by calculating the ratio between 7.35 µm counting beads and the number of events collected at the BCMP gate (approximately 0.6–0.9 mm). When the mixture was analysed by flowcytometry, each 7.35 µm counting bead formed a dot in the gate of the large-size population. If the number of total counting beads is 10,000, the number of BCMP can be calculated with formula: N=10,000 (BCMP%/Counting beads%).

Electron microscopy

Electron microscopy was performed as previously described [35]. Electron microscopy was conducted for the structural analysis. Accordingly, all BCMPs, HUVECs, and platelets were cross-sectioned and fixed on the glass coverslips. Fixation was conducted initially using 2.5% glutaraldehyde, and the same were stored at 4 °C, until further processing. At the time of processing, all the cover slips were washed with 0.1 M Na-cacodylate HCl buffer. The second fixation was carried out using 1% OsO_4, which was conducted before dehydration at a variable concentration of ethanol. An approximately 10nm thick layer of platinum was sprayed on the slides and the same were examined under the ultra-high-resolution mode of S-3400N Scanning Electron Microscope (Hitachi Ltd., Tokyo, Japan).

BCMPs were fixed with glutaraldehyde 2.5% in concentration for about 24 h at 4 °C. The slides were washed with 0.1 M cacodylate buffer and further fixed with 2% aqueous OsO_4 or can also be fixed with 0.2 M cacodylate for about 2 h at 4 °C. The slides were dehydrated and embedded in Epon 812 for about 2 h. Further to which, ultrathin sections of the samples were made and further treated with uranyl acetate for 2 h followed with lead citrate for 5 min. The slides were then heat dried and observed under TEM JEM-1400 by JEOL (Tokyo, Japan).

Platelet isolation from human blood

The peripheral blood drawn from the vein was initially spun at 200 g for about 15 min; to separate plasma from red blood cells. The separated plasma was further subjected to centrifugation at 1000 g for 10 min. The supernatant with poor platelet concentration was removed and the pellet high in platelet was washed with ACD/ HEPES Tyrode's buffer 1:8.3, v/v); and resuspended again with 1-2 mL of HEPES Tyrode's buffer. The final number of platelets was determined through Neubauer's chamber. The BCMPs/platelet interaction was evaluated using a standard curve of washed platelet and OD readings of final platelet suspension at 420 nm. The stock was adjusted to a final concentration of 1×10^6/mL for flow cytometric analysis.

Assays for extrinsic, intrinsic, FXa and prothrombinase activity

As described previously, the formation of extrinsic FXa, intrinsic FXa and prothrombinase was analyzed [47]. The determination of production of intrinsic FXa was carried using 1×10^8

cells/mL that was further incubated with 1 nM factor FIXa, thrombin (0.2 nM), 130 nM factor X, 5 nM factor VIII and 1.5 mM $CaCl_2$ in FXa buffer, prepared using TBS with 0.2% BSA at room temperature for 5 min. 7 mM EDTA was added as a stopping buffer. With the final concentration of 10 μL S-2765 (0.8 mM, final), the analysis of FSA was done in kinetic mode through automatic microplate reader (Tecan Infinite M200). The activation of extrinsic FXa was achieved with the help of factor X (130 nM), and addition of multiple other factors like 1 nM FVIIa as well as 1.5 mM $CaCl_2$. The determination of extrinsic FXa was done in a similar manner as with intrinsic FXa. Assessment of results was done against the rate of substrate cleavage of a standard dilution FXa.

For the production of thrombin, cells were incubated with 0.05 nM FXa as well as 1 nM Factor Va, $CaCl_2$ (1.5 mM) in prothrombinase buffer as well as 1 μM prothrombin at room temperature for 5 min. The rate of production of thrombin was measured using 10 μL S-2238 (0.8 mM, final), after the addition of stopping buffer EDTA at 405 nm on a kinetic microplate reader. With minor modifications, fibrin clots were evaluated as described previously [19]. In order to explain briefly, platelets that were isolated from the plasma were re-calcified at a final concentration of 1.5 mM $CaCl_2$ and pooled platelet-free plasma at a concentration of 86.7% of plasma. Whereas the turbidities at their highest levels of fibrin clots was calculated using the Tecan microplate reader.

Thrombin/anti-thrombin complexes

The analysis of thrombin and anti-thrombin complexes were carried using the Enzygnost TAT ELISA (Siemens Healthcare Diagnostics, Deerfield, IL, USA). The collected peripheral blood was centrifuged at 1250 g for 5 min, in order to separate plasma from platelets. The separated plasma had been aliquoted and stored further at -80 °C. The protocol had been designed as per instructions and final analysis was done with reference to the standard, which was supplied human TAT with known concentration.

Endothelium permeability assay

Endothelial cells were propagated on transwell polyester membranes with 3 mm pore size and 6.5 mm diameter at an average density of 2×10^5 cells/well (Costar, Corning, NY). Cells were grown to confluence until the 3rd day and then treated with BCMPs, SF or the supernatants from the last BCMPs washing. Growth medium containing 4% BSA (Gibco) was mixed with Evens blue (Sigma, St Louis, MO). At different time points, the permeability was measured by adding fresh medium-free BSA to the lower chamber and Evens blue BSA mixture to the upper chamber in each well. After 10 min, the optical density of Evens blue BSA in the lower chamber was measured using a fluorescence microplate reader at 650 nm.

Assay for endothelial cell barrier function

The transwell insert of permeability assay was conducted in order to determine the integrity of the endothelial cells barrier. For the same, around 20000 endothelial cells were seeded on the upper half of the transwell insert and allowed to be confluent that took around 2 days. The confluent monolayer of HUVECs was further treated with BCMPs at a concentration of 2.5 and/or 5.0×10^4/μL for about 8,16 as well as 24 h. The negative, as well as positive controls, were used as BCMP free groups as well as 0.5 mM EDTA-treated groups respectively. Further, 2×10^4 MDA-MB-231 cells were resuspended in a serum-free medium inside the upper chamber, and complete Endothelial cells with medium with 5% FBS was added in the lower

chamber for further incubation for overnight. Washing of transwell inserts was carried out using PBS and the cells from the upper chamber were removed using cotton buds. Cells that were migrated in the lower chamber were fixed using 4% paraformaldehyde and further stained with crystal violet stain. The cells were mounted on glass slides for further analysis. At least, three microscopic observations were carried out using for counting the number of migrated cells.

Supplementary Figures

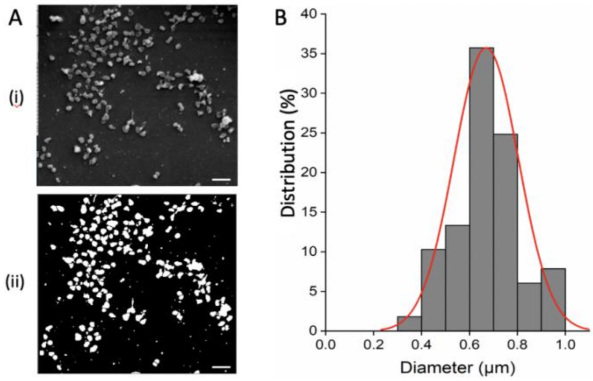

Figure S1. Representative Image J plots for the analysis of BCMPs diameter range. (A) Process map for determining particle sizes including i) the raw SEM image, ii) the black and white converted image, and the resulting segmented image. The diameter for each particle was computed from the area output by Image J under a spherical approximation. This process was repeated over multiple experimental trials (N = 5) for each formulation condition. Scale bars are 2 μm in length. **(B)** Resulting the range of BCMPs diameters.

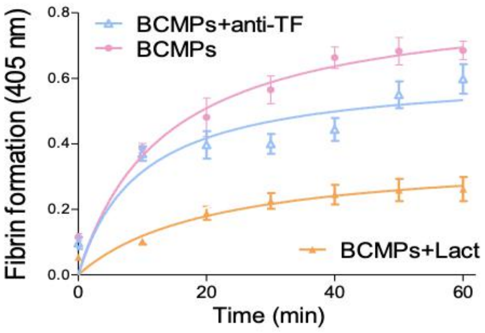

Figure S2. Lactadherin reverses the BCMPs-mediated procoagulation. Fibrin production cultured with BCMPs ($5.0 \times 10^4/\mu L$) was detected in the presence of recalcified microparticle-depleted plasma with or without lactadherin (128 nM) or anti-TF (25.6 μg/mL). Abbreviations: BCMPs, microparticles derived from breast cancer cells; Lact, lactadherin; TF, tissue factor.

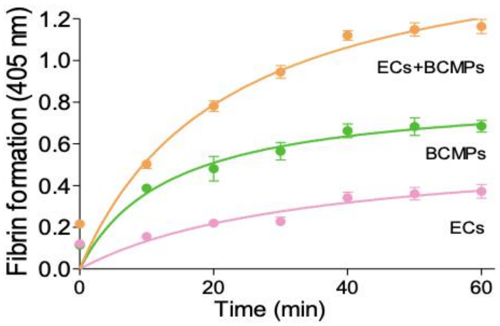

Figure S3. BCMPs transform endothelial cells into procoagulant phenotypes. Fibrin production cultured with different stimulation was detected in the presence of recalcified microparticle-depleted plasma. Abbreviations: BCMPs, microparticles derived from breast cancer cells; ECs, endothelial cells.

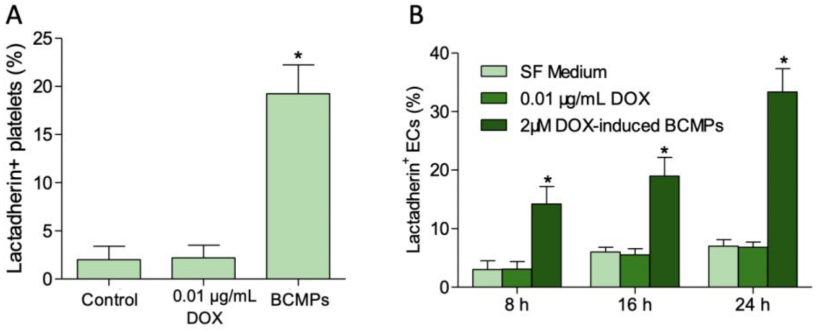

Figure S4. The activation of cells induced by different stimulation. The addition of BCMPs, generated from 2 μM DOX-treated MDA-MB-231 tumour cells, resulted in obviously discernible platelets (A) and ECs (B) activation, whereas the addition of 0.01 μg/mL DOX had no discernible effect. Statistics: Student t test. Data are mean ± SD. **P* < 0.001 vs. 0.01 μg/mL DOX. Abbreviations: DOX, Doxorubicin; ECs, endothelial cells; SF, serum free medium.

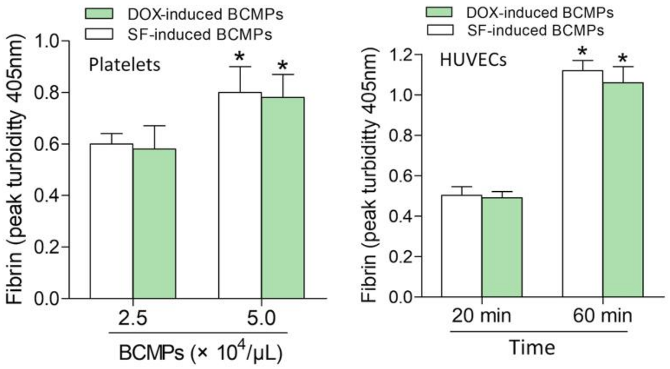

Figure S5. Fibrin production on DOX-induced or SF-induced BCMPs-cultured platelets or HUVECs was detected in the presence of recalcified MP-depleted plasma. SF-induced BCMPs and DOX-induced BCMPs have similar procoagulant activity. Statistics: Student t test. Data are mean ± SD. **P* < 0.01 vs. 2.5 ×10^4/μL BCMPs/20min. Abbreviations: DOX, Doxorubicin; ECs, endothelial cells; SF, serum free medium.
